# Role of the renin–angiotensin system in the pathophysiology of coronary heart disease and heart failure: Diagnostic biomarkers and therapy with drugs and natural products

**DOI:** 10.3389/fphys.2023.1034170

**Published:** 2023-02-23

**Authors:** Jinit K. Mehta, Ginpreet Kaur, Harpal S. Buttar, Hala Abubaker Bagabir, Rania Abubaker Bagabir, Sali Abubaker Bagabir, Shafiul Haque, Hardeep S. Tuli, Istvan G. Telessy

**Affiliations:** ^1^ Department of Pharmacology, Shobhaben Pratapbhai Patel School of Pharmacy and Technology Management, SVKM’s NMIMS, Mumbai, India; ^2^ Department of Pathology and Laboratory Medicine, Faculty of Medicine, University of Ottawa, Ottawa, ON, Canada; ^3^ Department of Physiology, Faculty of Medicine, King Abdulaziz University, Rabigh, Saudi Arabia; ^4^ Department of Hematology and Immunology, College of Medicine, Umm Al-Qura University, Makkah, Saudi Arabia; ^5^ Genetics Unit, Department of Medical Laboratory Technology, Faculty of Applied Medical Sciences, Jazan University, Jazan, Saudi Arabia; ^6^ Research and Scientific Studies Unit, College of Nursing and Allied Health Sciences, Jazan University, Jazan, Saudi Arabia; ^7^ Gilbert and Rose-Marie Chagoury School of Medicine, Lebanese American University, Beirut, Lebanon; ^8^ Centre of Medical and Bio-Allied Health Sciences Research, Ajman University, Ajman, United Arab Emirates; ^9^ Department of Biotechnology, Maharishi Markandeshwar Engineering College, Maharishi Markandeshwar (Deemed to be University), Ambala, India; ^10^ Department of Pharmaceutics, Faculty of Pharmacy, University of Pécs, Pécs, Hungary

**Keywords:** renin–angiotensin system, coronary heart disease (CAD), oxidative stress, diagnostic biomarkers, treatment of coronary heart disease

## Abstract

The renin–angiotensin system (RAS) plays a pivotal role in blood pressure regulation. In some cases, this steering mechanism is affected by various deleterious factors (mainly *via* the overactivation of the RAS) causing cardiovascular damage, including coronary heart disease (CHD) that can ultimately lead to chronic heart failure (CHF). This not only causes cardiovascular disability and absenteeism from work but also imposes significant healthcare costs globally. The incidence of cardiovascular diseases has escalated exponentially over the years with the major outcome in the form of CHD, stroke, and CHF. The involvement of the RAS in various diseases has been extensively researched with significant limelight on CHD. The RAS may trigger a cascade of events that lead to atherosclerotic mayhem, which causes CHD and related aggravation by damaging the endothelial lining of blood vessels *via* various inflammatory and oxidative stress pathways. Although there are various diagnostic tests and treatments available in the market, there is a constant need for the development of procedures and therapeutic strategies that increase patient compliance and reduce the associated side effects. This review highlights the advances in the diagnostic and treatment domains for CHD, which would help in subjugating the side effects caused by conventional therapy.

## Introduction

Cardiovascular diseases (CVDs) are characterized by multifaceted abnormalities represented by the inability of the heart to pump sufficient blood to meet different biological/biochemical and oxygen needs of the body at rest or during exercise. The renin–angiotensin–aldosterone system (RAAS) plays an important role in regulating the systolic and diastolic blood pressure in the body, whereas its overactivity leads to a cascade of deleterious changes in the cardiovascular and renal system and causes endothelial dysfunction of the arterial blood vessels. Several pathological conditions such as atherosclerosis, hypertension, diabetes, severe anemia, and anti-cancer drug therapy culminate in causing cardiac dysfunction leading to coronary heart disease (CHD), chronic heart failure associated with pressure overload, volume overload or myocardial infarction (MI), and ischemic heart disease (Perazella and Setaro, 2003). The various clinical signs of a failing heart include shortness of breath, lung congestion, fluid retention, exercise intolerance, weakness, fatigue, and peripheral edema, which are used for the diagnosis of heart failure. It should be emphasized that a wide variety of mechanisms are also associated with it (McIlvennan and Allen, 2016).

CVDs continue to remain the leading cause of morbidity and mortality all over the world. A wide array of mechanisms are associated with CVDs, such as atherosclerosis, hypertension, valvular heart disease, coronary heart disease (CHD), thrombogenesis, stroke, and chronic heart failure. The treatment of CVDs imposes an excessive economic burden on the society and healthcare systems globally (Thomas et al., 2018). The conventional risk factors of CVDs consist of atherosclerosis, hypertension, hyperlipidemia, hyperglycemia, and obesity. The lifestyle factors including tobacco smoking, a sedentary lifestyle and lack of exercise, unhealthy dietary habits, and a low socioeconomic status contribute heavily to the development of obesity, diabetes mellitus, and CVDs in children and adults. Sugar-loaded beverages and excessively salted foods are also potential risk factors. Atherosclerosis and atherosclerotic plaque formation, hypertension, obesity, and diabetes are the main cardiovascular risk factors that are directly correlated with unhealthy dietary practices and lifestyle (Francula-Zaninovic and Nola, 2018). It is now recognized that the best cost-effective methods for maintaining good cardiovascular health are heart healthy diets and an active lifestyle. Scientific research has established a strong link between antioxidant and anti-inflammation food choices, exercise, and smoking cessation in the maintenance of a healthy cardiovascular function (Yu et al., 2016). The early diagnosis of atherosclerosis and CVDs with the help of different diagnostic biomarkers assists in the prevention of CVDs, and management with drugs, dietary interventions and plant-derived therapies, regular exercise (30 min/day), and smoking cessation is the main issue discussed in this review.

**FIGURE 1:**
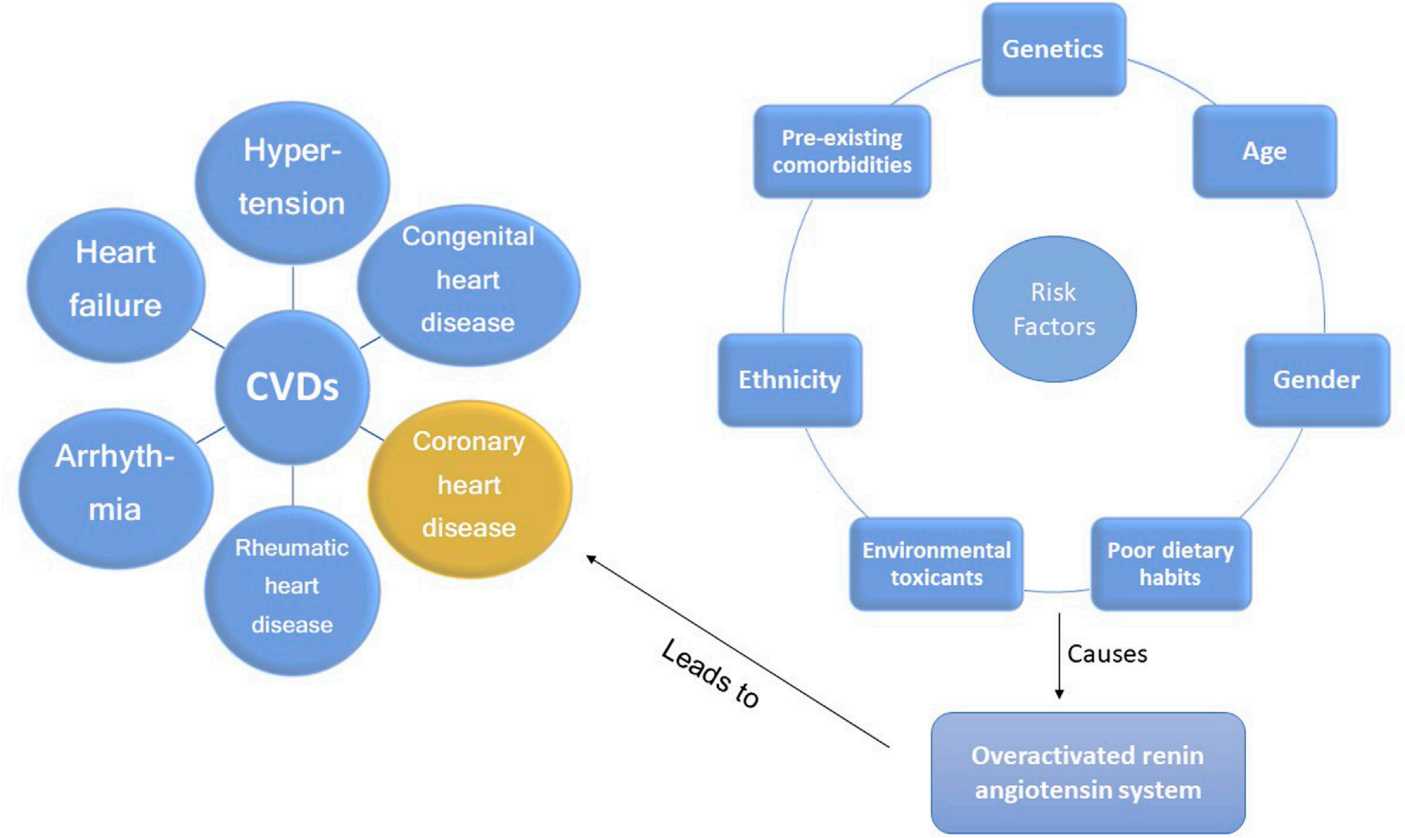
Risk factors that can accelerate the development of coronary heart disease *via* the overactivated RAS.

Different types of CVDs and risk factors involved in the progression of CHD are depicted in Figure 1. The various risk factors include genetics (Khera and Kathiresan, 2017), age (Madhavan et al., 2018), gender (Davies and Rier, 2018), poor dietary habits (Willett, 2012), environmental toxicants (Bhatnagar, 2017), ethnicity (Gaziano et al., 2010), and pre-existing co-morbidities.

Atherosclerosis and thrombosis in the coronary arteries are the most frequent causes of ischemic heart disease. Atherosclerosis involves the deposition of LDL-cholesterol, foam cells, and the infiltration of macrophages and white blood cells in the endothelium of arterial blood vessels. Atherosclerosis-induced stiffness of coronary blood vessels leads to hypoxia and reduced oxygen and nutrient supply to the myocardium. The progressive narrowing and stiffening of the coronary arteries, subsequently, provokes myocardial ischemia and the imbalance between the blood supply and energy demand to the myocardium (King, 1959). Epidemiological data collected from 1990 to 2017 showed that over 126 million people died worldwide as a result of CHD-related illnesses (Willett, 2012; Davies and Rier, 2018). If CHD is not diagnosed and treated on time, it could develop into acute heart failure (HF) or chronic heart failure (CHF), low cardiac output, and complex conditions in the kidney and other vital organs of the body.

### The role of the renin–angiotensin–aldosterone system

**FIGURE 2:**
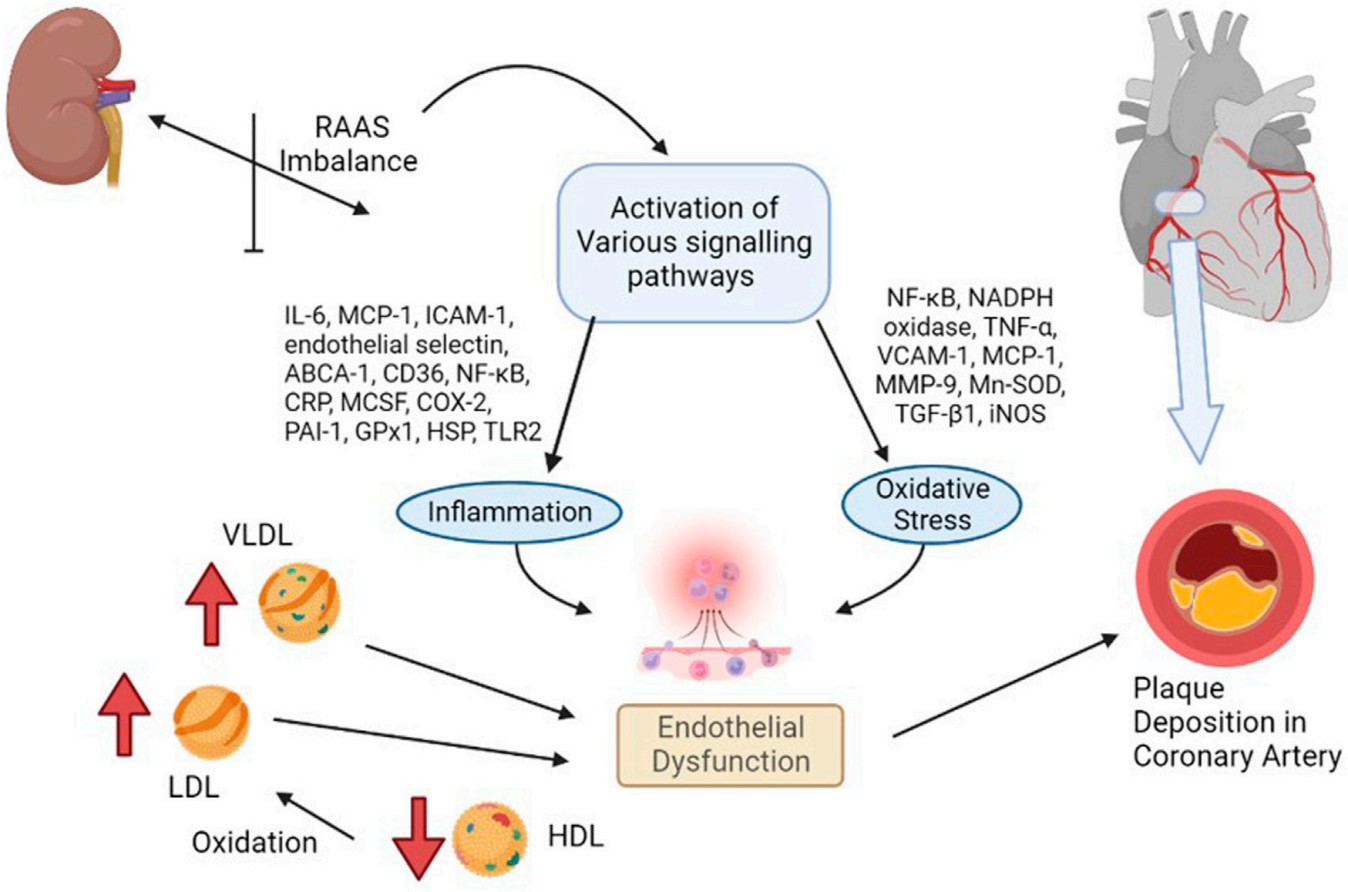
Renin–angiotensin system in the pathophysiology of coronary heart disease [overactivated renin–angiotensin system causes the activation of various harmful signaling pathways of inflammation and/or oxidative stress, or the harmful lipid molecules cause endothelial dysfunction, which exacerbates the process of coronary heart disease].

The RAAS is a blood pressure regulatory system within the cardiorenal unit, with direct action to the arterioles (arterial blood pressure) and additional action to the adrenal cortex (vascular volume + arterial blood pressure). The renin–angiotensin system (RAS), as part of the RAAS without the effect of aldosterone, plays a central regulatory mechanism, amongst others, of the extracellular volume and cardiovascular system. Apart from the normal physiological functions of the renin–angiotensin system (Wong, 2021), its overactivation leads to a cascade of inflammatory and oxidative stress processes, as illustrated in Figure 2, both of which contribute to cardiovascular diseases (Poznyak et al., 2021).

Furthermore, the inappropriate activation of the RAS has many deleterious effects, such as the pro-atherogenic potential, endothelial injury, insulin resistance, pro-thrombotic effect, and vascular smooth muscle cell and monocyte proliferation. The RAS promotes the progression of CHD by its interplay with angiotensin-II, which acts on vascular cells *via* direct and indirect mechanisms and on the upregulation of reactive oxygen species (ROS) and the concomitant downregulation of endothelial nitric oxide (NO) (Sheppard and Schiffrin, 2013) (Husain et al., 2015). Due to numerous drug-related adverse effects (Institute for Quality and Efficiency in Health Care (IQWiG), 2017) and gut dysbiosis (Weersma et al., 2020), the existing techniques for treating CHD caused by a hyperactive RAS have considerable disadvantages. As a result, novel, safe, and effective therapeutic pharmacotherapies targeting the RAS and the discovery of molecular biomarkers to detect the course of CHD in its early stages are required. Moreover, if CHD has been diagnosed, its progression should be followed in order to prevent and/or treat secondary HF. Here, the prevention usually contains medication effective in the RAAS, e.g., ACEi, ARB, or aldosterone antagonists, by which the development of CHF can be delayed.

### Diagnostic biomarkers

As the early detection of cardiovascular alterations offers a greater chance to effective interventions, the diagnostics play a crucial role in proper therapy. There are several diagnostic techniques to discover threatening tendencies in physiological parameters. One of them is a group of biological markers, whose research is still a hot topic (Dhingra and Vasan, 2017) (Jacob and Khan, 2018). Novel biomarkers for diagnosing CHD have become essential in the wake of non-specific biomarkers leading to delays in the exact diagnosis and subsequent mishap in the correct treatment regimen. Notably, the early diagnosis of CHD and its treatment provide a chance to prevent HF. Therefore, newer and specific biomarkers need to be studied for an early and accurate disease diagnosis. Along with the specificity, these biomarkers should be reliable, reproducible, and quantifiable. The upcoming biomarkers studied for CHD have been listed as follows:

A. Fibroblast growth factor 23 (FGF23)—It is a protein that aids in the metabolism of vitamin D and phosphate and sodium and calcium reabsorption from the kidney (Erben, 2018). Recent studies have co-related this protein with CHD. In a meta-analysis of eight studies, including 16,702 patients conducted by Zheng et al. points to a potential role of adverse cardiovascular outcomes in CHD patients (Zheng et al., 2022). A study by Hu et al. not only throws light on the independent co-relation of FGF23 with CHD but also gives an indication about the number of stenotic vessels proportional to FGF23 serum levels (Hu et al., 2015), which is in line with the studies of Xiao and collaborators (Xiao et al., 2013). Furthermore, greater serum concentrations of FGF23 were found to be linearly associated with the total atherosclerosis burden (Mirza et al., 2009), heart failure, and also, an all-cause mortality (Udell et al., 2014) (Parker et al., 2010).
B. Trimethylamine N-oxide (TMAO)—TMAO produced by the gut microbiota in body has been directly co-related with atherosclerotic heart disease (Wassenaar et al., 2021) (Schiattarella et al., 2017) (Stubbs et al., 2016). It is known to increase vascular inflammation by activating NF-κB (Seldin et al., 2016) and ROS–TXNIP–NLRP3 inflammasome (Sun et al., 2016) signaling pathways. It causes reverse cholesterol transport inhibition, variation in the bile acid, aggravates the inflammation of fat tissues, and changes the macrophage characteristics by downregulating the expression of the CYP7A1 enzyme necessary for bile acid synthesis and cholesterol breakdown and for the upregulation of the scavenger receptor A and CD36 (Liu et al., 2019). All these processes together contribute to the formation of foam cells (Geng et al., 2018).
C. MicroRNA (miRNA)—This small non-coding RNA regulates gene expression by identifying a defective mRNA and causing its downregulation when it is detected at the cell transcription level (Jungers and Djuranovic, 2021). Many miRNAs have been observed to have been modified during CHD, and miRNA-21 (Economou et al., 2015), miRNA-92a (Loyer et al., 2014), miRNA-106a-5p (Hu et al., 2020), miRNA-451a (Wang et al., 2021), miRNA-100 (Soeki et al., 2015), and miRNA-126 (Wang et al., 2017) are just a few of the many known miRNAs whose levels are disrupted during CHD.
D. Pentraxin 3 (PTX3)—Compared to the C-reactive protein, it is an independent biomarker and is expressed early in the endothelial vasculature inflammation making it a better and reliable biomarker for CHD (Wang et al., 2014) (Chu et al., 2019). It has also been known to increase the oxidation of LDL molecules, thereby causing accumulation (Casula et al., 2017). It is also known to cause oxidative insults to the endothelium by generating reactive oxygen species (Zlibut et al., 2019). The Bruneck Study confirmed the role of PTX3 in the late stages of atherosclerosis but not in acute phase reactions (Knoflach et al., 2012).
E. Myeloperoxidase—It is synthesized by macrophages only after there is an inflammatory insult and local cytokine activation in conditions such as atherosclerotic CHD. This inflammatory insult causes the increased transcription of the myeloperoxidase gene (Kumar et al., 2004) (Nauseef, 2018). Myeloperoxidase-containing macrophages were also found in susceptible and ruptured atherosclerotic plaques (Sugiyama et al., 2001). It destabilizes the plaque by the formation of oxLDL, which through a cascade of events decreases NO production and increases VCAM-1 recruitment *via* the modified HDL, which together cause endothelial injury (Ndrepepa, 2019).

### Therapeutic options

The guidelines on coronary artery disease recommend disease-specific combination therapies with the attention on concomitant diseases such as hypertension, dyslipidemia, diabetes, and kidney injury (Nakano et al., 2022) (Arnett et al., 2019). In case of hypertension, RAS-inhibitors are the leading drugs.

#### Pharmacological agents

The inhibition of the overactivated RAS results in the lowering of the blood pressure. Today, this is the most used group of antihypertensive products. The RAS-inhibition therapy can, currently, be divided into four ways according to their mechanism of action (Table 1). They are given in brief as follows:

i. The decrease of the renin release from the kidneys (e.g., beta-blockers)
ii. The inhibition of the effect of renin on angiotensin-I production (direct renin inhibitors, such as aliskiren)
iii. The inhibition of the effective angiotensin-II production from the inactive angiotensin-I (angiotensin-converting enzyme inhibitors (ACEis), such as enalapril)
iv. The inhibition of the binding of angiotensin-II to the angiotensin-II receptor (angiotensin-II receptor antagonists (ARBs), such as losartan)

**TABLE 1:** Synthetic drugs affecting the renin–angiotensin system and adverse effects caused by the prophylactic treatment leading to reduced patient compliance.

Type of drug	Main effect	Main adverse effect
Beta-blocker	Inhibition of renin release	Bradycardia
	Decreases the sympathetic tone of CNS	Increases LDL-C level and decreases HDL-C level
	Reduces cardiac volume	Rebounds hypertension
Ri	Blocks the angiotensinogen (AG) receptor, which inhibits the angiotensin-I (AT-I) conversion by blocking the binding of renin to AG	Persistent cough, hyperkalemia, severe hypotension, angioedema, arthralgia, and diarrhea
ACEi	Decreases the blood and tissue concentration of angiotensin-II by blocking angiotensin-1 for angiotensin-2 conversion	Cough due to the bradykinin and substance-P release; contraindicated in pregnancy
ARB	Selectively blocks angiotensin-2 receptors resulting in the inhibition of vasoconstriction	Hyperkalemia
NEPi	Blocks the decomposition of natriuretic peptides	Angioedema and arrhythmia

RAS, renin–angiotensin system; CNS, central nervous system; LDL-C, low-density lipoprotein fraction of cholesterine; HDL-C, high-density lipoprotein fraction of cholesterine; Ri, renin inhibitor; ACEi, angiotensin-converting enzyme inhibitor; ARB, angiotensin-II receptor blocker; NEPi, neprilysin inhibitor.

In addition to RAS-inhibitors, neprilysin inhibitors that block both the neprilysin (the zinc-metalloprotein enzyme that inhibits the degradation of the three vasodilator natriuretic peptides) and angiotensin-II breakdown are used alone or in combination with RAS-inhibitors (ARNI = angiotensin receptor–neprilysin inhibitor, e.g., omapatril as the non-registered single molecule and the more successful sacubitril–valsartan as a registered combination). This type of therapy reduces the blood pressure and heart failure hospitalization (Sutanto et al., 2021).

Table 1 depicts the various adverse effects of synthetic therapeutics; therefore, natural remedies are a topic of research interest. Table 2 provides an insight into various natural therapies that are, currently, under research as potential cardiovascular drugs or drug adjuncts.

B. Dietary foods—The diet of people in everyday life is the biggest source of medicine provided the correct food type and optimum amounts are consumed. Therefore, exploring healthy options is the need of the hour and designing a nutrient rich diet is required to keep diseases at bay.
B1. Pomegranate—All the components such as seeds, flowers, fruits, and leaves possess various pharmacological properties. Pomegranate juice has been shown to reduce cellular oxidation and increase paraoxonase 2 activities in the peritoneal macrophage (Rosenblat et al., 2015). In the context of the topic of review, the juice at a concentration of 100 mg/kg/day and 300 mg/kg/day in Wistar rats was also found to inhibit ACE rendering it non-active alongside the antioxidant activity to counter the angiotensin-II activity (Mohan et al., 2010).The dry skin extract also showed significant improvements in endothelial dysfunction with the activation of the protein kinase B/eNOS signaling and reducing vasculitis (Vilahur et al., 2015). Another hydroethanolic extract of the peel showed improvement in the plaque necrosis phase of CHD along with improvements in harmful lipid parameters and systemic inflammation (Manickam et al., 2022).
B2. Dark chocolate—Studies have shown the improvement of endothelial dysfunction *via* the inhibition of LDL-cholesterol, oxLDL, and triglycerides, while increasing the HDL-cholesterol. It leads to a significant inhibition of ACE after 3 h of consumption (Persson et al., 2011).
B3. Artichoke—A study by Lupattelli et al. showed the positive effects of artichoke juice on imbalanced cholesterol levels and humoral markers such as LDL, total cholesterol, VCAM-1, and ICAM-1, respectively, which are the causal agents of endothelial dysfunction (Lupattelli et al., 2004). In another study by Küçükgergin et al., the artichoke leaf extract also provided the same results as the artichoke juice along with good antioxidant activities (Küçükgergin et al., 2009).
B4. Garlic—It reduces the total LDL, total cholesterol, and triglyceride with an effect on HDL and creatine kinase; it protects eNOS from being degraded by the protein kinase B signaling pathway, thus protecting the endothelial lining from the oxLDL insult. It also slows the progression of the necrotic plaque area with foam cells (Knox and Gaster, 2007) (Lei et al., 2010) (Sobenin et al., 2019). The aqueous extract showed reversed effects on increased ACE levels, which might lead to the expression of the protective angiotensin (Perazella and Setaro, 2003; McIlvennan and Allen, 2016; Yu et al., 2016; Khera and Kathiresan, 2017; Francula-Zaninovic and Nola, 2018; Madhavan et al., 2018; Thomas et al., 2018) (Mahmoud et al., 2014).
C. Antioxidants—These molecules assist in trapping reactive oxygen species and reducing the amount of free radicals in the body to protect macromolecules such as VLDL and LDL from causing damage, thereby reducing endothelial dysfunction. A lot of publications revealed a very close correlation between oxidative stress and the RAS overexpression (Fanelli and Zatz, 2011). The increase of ROS levels and NADPH oxidase expression can be prevented by the administration of, e.g., angiotensin-1 receptor antagonists. Thus, the role of the RAS in this regard and the reason for attacking its pathways is clear.
C1. Catechins—Tea beverages have been consumed since ages. Green tea catechins have been shown to decrease cholesterol and triglyceride levels and increase fat excretion, which synergistically downregulates the plaque formation (Khan and Mukhtar, 2013). It also led to the AMPK pathway activation and the PKA-dependent pathway, which led to the upregulation of fatty acid oxidation and lipolysis, respectively (Chen et al., 2020). In addition to lipid levels, a study by Tu et al. has been shown to decrease inflammatory markers such as TNF-α, C-reactive protein, and IL-6 (Tu et al., 2018). Additionally, black tea contains a very potent renin inhibitor named theasinensin B (Li et al., 2013), and a thorough literature search revealed no further testing of this compound in any particular cardiovascular complications, thereby making it a potential candidate for testing.
C2. Vitamin E—Vitamin E administered in combination with vitamin C has shown to mitigate risk factors associated with atherosclerotic progression. It has shown little effect on endothelium-dependent vasodilation to improve blood flow in obstructive CHD (Uzun et al., 2013). It prevented HDL remodeling, improved antioxidant activity, and reduced the total cholesterol, triglyceride, and TNF-α level (Contreras-Duarte et al., 2018).
C3. Alpha lipoic acid (ALA)—It is a molecule causing the free radicals to become inactivated, while the reduced form binds ROS (Packer and Cadenas, 2011). This is highlighted by various studies proving the antioxidant potential of this molecule by decreased serum levels of malondialdehyde and increased superoxide dismutase levels (Han et al., 2018). Additionally, it is also proved to be a prospective hypolipidemic by reducing the levels of triglycerides in the body (Agathos et al., 2018). It also modulates the NF-κB signaling pathway (Gomes and Negrato, 2014), improves endothelial dysfunction (Wray et al., 2012), and inhibits atherosclerotic plaque formation (Ying et al., 2010).
C4. Lycopene—It is suggested to prevent the formation of oxLDL at the early stages, which might help in slowing the progression of CHD (Pennathur et al., 2010) (Zuorro et al., 2013). It is a powerful singlet oxygen quencher and downregulator of ROS as proved in the studies by Liu and others using angiotensin-II as the inducer (Liu et al., 2015). It is also known to inhibit TNF-induced NF-κB activation (Prasad et al., 2014) and reduce VCAM-1 and LDL concentrations (Sanajou et al., 2018).
D. Phytotherapies—The bioactive compounds from fruits, vegetables, and medicinal and aromatic plants are the main acting functional moieties that have led to extensive research to exploit their therapeutic uses for different diseases.
D1. Allicin—It has been shown to decrease homocysteine levels, which is an important risk factor for CHD. It also improves the impaired endothelial function due to hyperhomocysteinemia and decreases total cholesterol and triglyceride levels, which are under tight control of the RAS (Esse et al., 2019) (Li et al., 2020) (Liu et al., 2017). It also has been shown to have attenuated angiotensin-II levels in a rat CKD model making it a useful treatment adjunct (García-Trejo et al., 2016). A study by Oktaviono et al. showed that allicin combined with vitamin C shows a dose dependent increase in the migration of endothelial progenitor cells, which are responsible in repairing the endothelial dysfunction caused by various stresses to the membrane (Oktaviono et al., 2020) and the RAS (Amraei and Rahimi, 2020).
D2. Colchicine—A rat model of atrial fibrillation revealed the action of colchicine on renin inhibition, indicating its potential use in CHD treatment (Yue et al., 2019). It has also been shown to inhibit the NLRP3 inflammasome pathway, decrease thromboxane A2, leukotriene B4, and cyclo-oxygenase 2 levels, and increase prostaglandin E2 production (Butt et al., 2021). Colchicine is a potent antagonist of the RAS and has the potential to become a new standard therapy for the prevention of CHD (Yong et al., 2022). A meta-analysis by Abrantes et al. also pointed toward the adverse gastrointestinal effects of daily colchicine consumption in addition to the cardioprotective effects (Abrantes et al., 2021), making it necessary to administer the gastroprotective adjunct along with colchicine.
D3. Betalain—Betalain supplementation decreases the concentration of the total cholesterol, triglyceride, and LDL-cholesterol, which slows atherosclerotic plaque formation. As angiotensin-2 induces plaque formation at an early stage, betalain may antagonize this type of progression. It also reduces intercellular and vascular cell adhesion molecules along with interleukin 6, TNF-α, and endothelial-leukocyte adhesion molecule 1 (Rahimi et al., 2019a) (Rahimi et al., 2019b). Even with these findings, the exact effects of betalain (both betacyanin and betaxanthin) need further mechanistic studies of the exact effects on the RAS.
D4. Resveratrol—Mice studies have shown protective effects of resveratrol by its various actions on RAS components such as the increased eNOS and AT2R/Ang 1–7/MasR axis expression and ACE2 levels with a simultaneous decrease in ACE, angiotensin-II, and AT1R. Certain inflammatory and oxidative stress parameters were suppressed (Jang et al., 2018). A plausible mechanism of its protective action is that it is mediated by the antioxidant enzyme heme oxygenase-1 and NO. It also inhibits cardiac cell death and increases the production of the vascular endothelial growth factor. Preclinical research suggests a large dose to avoid necrotic region expansion and improve heart function. Clinical investigations have shown that increasing adiponectin and inhibiting the thrombogenic plasminogen activator inhibitor type 1 protects the heart (Pagliaro et al., 2015) (Salehi et al., 2018) (Buttar et al., 2005).

**TABLE 2:** Natural products (dietary foods, phytotherapeutics, and antioxidants) as therapeutic strategies to target various inflammatory and oxidative stress parameters of coronary heart disease.

Category	Name	Major bioactive molecule	Source
Dietary foods	Pomegranate	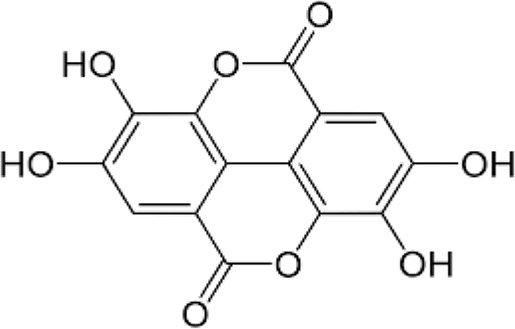	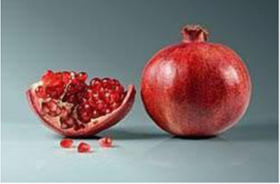
	Dark chocolate	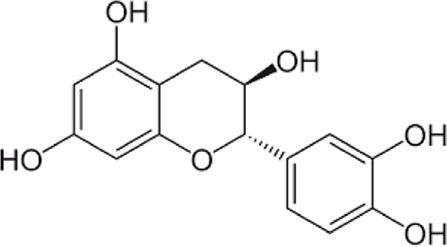	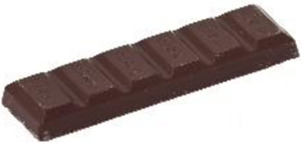
	Artichoke	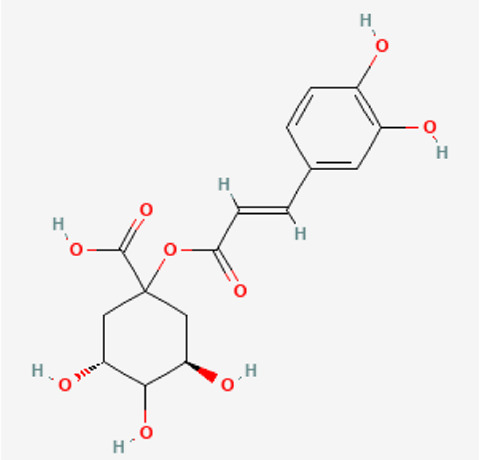	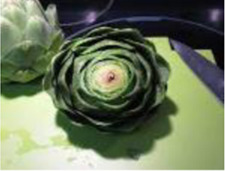
Antioxidants	Catechins	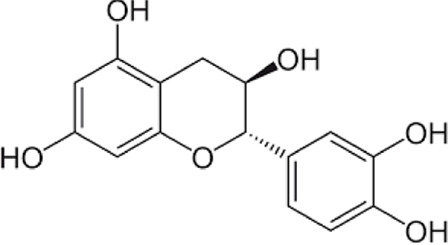	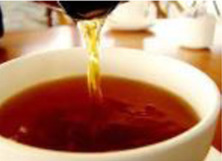 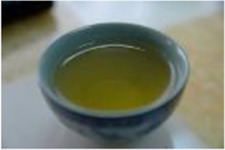 Black tea and green tea
	Vitamin E	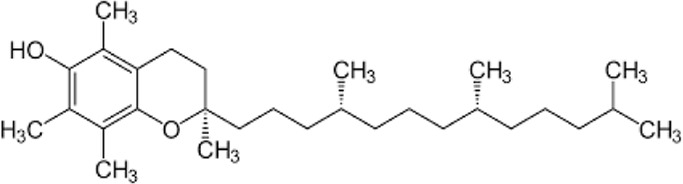	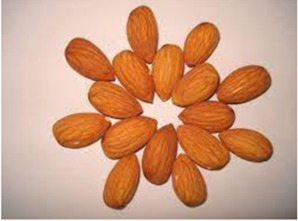 Almond
	Alpha lipoic acid	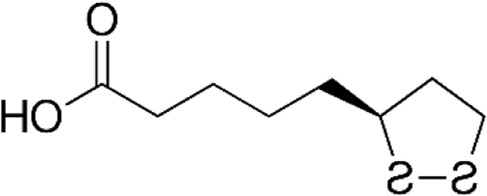	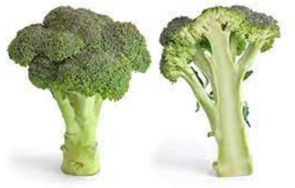 Broccoli
	Lycopene	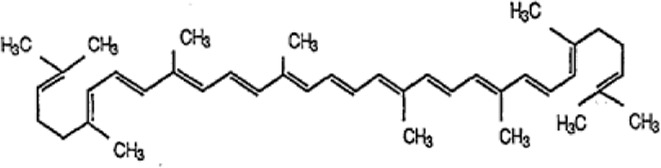	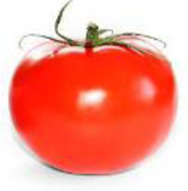 Tomato
Phytotherapies	Allicin	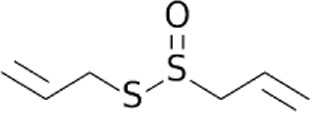	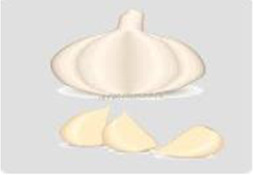 Garlic
	Colchicine	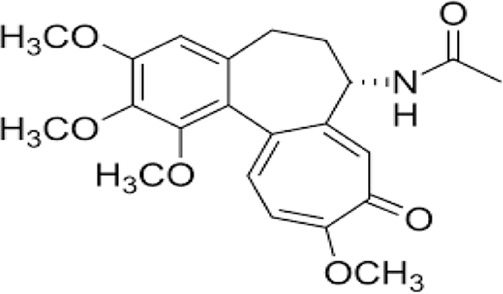	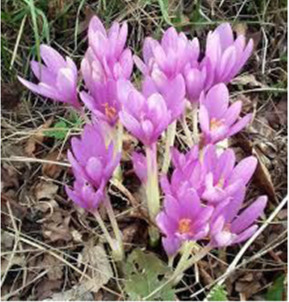 Colchicum autumnale
	Betalains	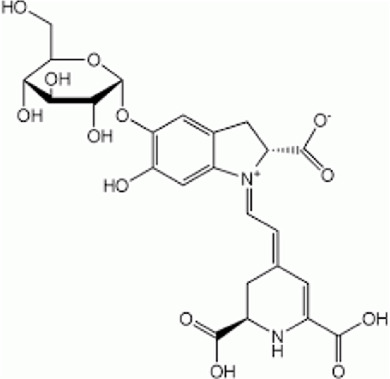	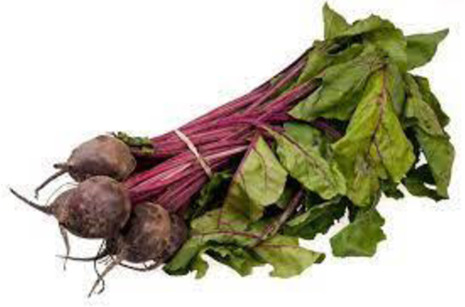 Beetroot
	Resveratrol	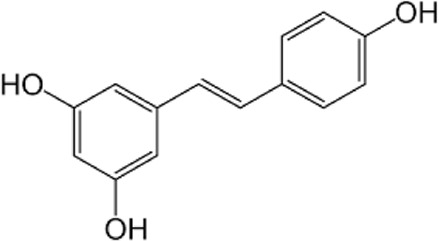	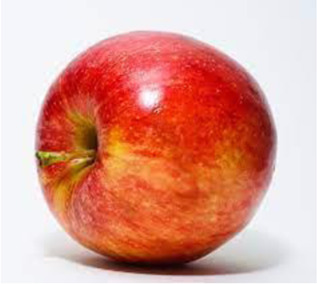 Apple

## Concluding remarks and future strategies

The focus of this review was to evaluate the deleterious effects of the overactive RAS on the cardiovascular system and how it influences the occurrence of CHD and chronic heart failure when underdiagnosed and left untreated. The conventional synthetic therapeutics are known to cause adverse outcomes in long-term prophylaxis leading to patient discomfort and reduced compliance. The discovery of cost-effective new therapies is driven by the need to eliminate these adverse consequences associated with current treatment options. More emphasis is being placed on naturally derived remedies, which can help prevent undesirable side effects in most cases. Such alternative treatments also relieve the medical community of a significant amount of burden, provided they are prescribed as a part of routine practice. As a result of these combined benefits, the patient compliance improves. Therefore, exploring new breakthrough therapies is mandatory to control the number of mortalities and morbidities associated with cardiovascular complications and prevent CHD to progress to fatal chronic heart failure and negate the harmful effects of the RAS. This review has tried to bridge some of the gaps associated with the unawareness of natural remedies, which have been successfully studied and can be used as alternatives to conventional therapies.

The results of epidemiological, experimental, and clinical studies have unequivocally demonstrated the positive impact of physical activity, antioxidant/anti-inflammation dietary interventions (e.g., intake of fresh fruits, vegetables, probiotics, fibrous foods, and omega-3 polyunsaturated fatty acids), obesity and diabetes reduction, and the cessation of cigarette smoking for improving the cardiovascular health and prevention of CVDs. The prophylactic measures must be dealt with collectively because there is overwhelming evidence that the occurrence of CVDs can be reduced by approximately 80% by making lifestyle modifications. Furthermore, the preventive strategies against CVDs must be targeted at the primary health promotion level before some of the important underlying causes of CVDs seriously afflict a person or a population at large. Such cost-effective preventive approaches will help in reducing not only CVDs and employee absenteeism but also the hospital and drug costs burdening healthcare systems of both developed and developing countries.

## Abbreviations

CHD, coronary heart disease; CVD, cardiovascular diseases; CHF, chronic heart failure; RAAS, renin–angiotensin–aldosterone system; RAS, renin–angiotensin system; ACEi, angiotensin-converting enzyme inhibitor; ARB, angiotensin-2 receptor blocker; NO, nitric oxide; FGF23, fibroblast growth factor 23; TMAO, trimethylamine N-oxide; miRNA, microRNA; PTX3, pentraxin 3; VCAM-1, vascular cell adhesion molecule 1; ARNI, angiotensin receptor–neprilysin inhibitor; VLDL, very-low-density lipoprotein; LDL, low-density lipoprotein; oxLDL, oxidized low-density lipoprotein; HDL, high-density lipoprotein; TNF-α, tumor necrosis factor *α*; ACE, angiotensin-converting enzyme; AT1R, angiotensin-II type 1 receptor; AT2R, angiotensin-II type 2 receptor; CKD, chronic kidney disease.
